# Changes of the Fatty Acid Profile in Erythrocyte Membranes of Patients following 6-Month Dietary Intervention Aimed at the Regression of Nonalcoholic Fatty Liver Disease (NAFLD)

**DOI:** 10.1155/2018/5856201

**Published:** 2018-12-04

**Authors:** Dominika Maciejewska, Wojciech Marlicz, Karina Ryterska, Marcin Banaszczak, Dominika Jamioł-Milc, Ewa Stachowska

**Affiliations:** ^1^Department of Biochemistry and Human Nutrition, Pomeranian Medical University, Szczecin, Poland; ^2^Department of Gastroenterology, Pomeranian Medical University, Szczecin, Poland

## Abstract

**Background:**

Nonalcoholic fatty liver disease (NAFLD) is closely related to the metabolism disorders of fatty acids. The pathogenesis of the disease includes an increased concentration of FFA in blood, an increase in the biosynthesis of fatty acids, and disorders in the process of *β*-oxidation.

**Objective:**

The aim of the study was to analyze the fatty acids in erythrocyte membranes among 55 patients with NAFLD who were subjected to a 6-month dietary intervention in order to reduce fatty liver.

**Materials and Methods:**

Basic anthropometric and biochemical measurements were performed. The profile of fatty acids was measured in the membranes of erythrocytes and analyzed by gas chromatography. The dietary compliance was evaluated using 72-diary questionnaires, anthropometric measurements.

**Results:**

With the reduction of fatty liver (p<0.01), the patients' biochemical and anthropometric parameters were significantly improved. A significant decrease in the concentration of alanine aminotransferase (p<0.01) and asparagine aminotransferase (p<0.01) was observed, along with a decrease in the amount of insulin (p<0.05) and insulin resistance (p<0.05). Significant changes in terms of the fatty acid profile were observed among patients who followed the dietary intervention. There was a noticeable tendency in terms of the reduction palmitic acid (p<0.055) and a significant reduction of stearic acid (p<0.05). Significant changes in the profile of fatty acids were also associated with the reductionof palmitoleic (p<0.05) and oleic acids (p<0.05). Another statistically significant change observed was the increase in polyunsaturated fatty acids. In particular (p<0.01) the rise of eicosapentaenoic (p<0.055) and docosahexaenoic acids (p<0.55) was noted.

**Conclusion:**

The profile of fatty acids turned out to be a potential biomarker of the liver changes during NAFLD regression. Further research is needed to fully elucidate the usefulness and applicability of our findings in the management of NAFLD.

## 1. Introduction

The term “nonalcoholic fatty liver disease” (NAFLD) was first introduced by Ludwig in 1980. He described NAFLD as a liver disease based on the accumulation of lipids in the hepatocytes of people who did not overuse alcohol and consumed less than 20 g of ethyl alcohol per day. NAFLD encompasses a variety of disorders that include simple steatosis without the symptoms of hepatocyte damage, but also active steatosis with an ongoing inflammation and the progression to cirrhosis. The prevalence of this disease in the United States of America is estimated at about 20-40% [1, 2], whereas in the European population the value is between 2 and 44% [3]. The pathogenesis and development of NAFLD is a complex process and involves multiple factors, such as dyslipidemia, insulin resistance, mitochondrial dysfunction, oxidative stress, the development of an inflammation, disorders in the metabolism of adipose tissue, microbiota alterations, and genetic factors. Because the pathogenesis of this disease is multifactorial, the generally accepted and new approach to this pathological unit is the multiple hits hypothesis. In physiological conditions, lipids accumulated in the liver should constitute about 3-5% of the liver's mass. In the case of simple fatty liver, the amount of hepatocytes with fat accumulated within the cells (fat consisting mostly of triacylglycerols (TG)) exceeds 5% [4–6]. The illness also manifests itself in the form of biochemical disorders related to the changes in the metabolism of carbohydrates and fats [1].

NAFLD is closely connected to the metabolic disorders of fatty acids. The pathogenesis includes an increased concentration of free fatty acids (FFA) in blood and increased biosynthesis of fatty acids in the liver, as well as disorders related to the process of *β*-oxidation. The increased level of fatty acids is a significant factor leading to fatty liver. Donelly et al. showed that about 60% of triglycerides present in the liver come from the absorbed unesterified fatty acids [7]. Patients diagnosed with fatty liver often have an increased concentration of plasma FFA, which is associated with the increased release of acids in the process of adipose tissue lipolysis [8–10]. Additionally, the process is sustained due to the peripheral insulin resistance [11]. Another important aspect of fatty acid metabolism disorders is the* de novo *lipogenesis in the liver. TG originating from the* de novo *lipogenesis (DNL) physiologically do not exceed the level of 5% of secreted VLDL. Donnelly et al. showed that the involvement of TG originating from the* de novo* synthesis of fatty acids is at the level of up to 30% of synthesized TG [7]. DNL is related to the increased activity of fatty acid synthase (FAS), elongase 6 (ELOVL 6), and stearoyl-CoA desaturase (SCD). These enzymes are strictly regulated by groups of transcription factors. Three, most important families of transcription factors regulating these processes could be distinguished: sterol regulatory element binding protein (SREBP), liver X receptors, (LXR), and carbohydrate response element binding protein (ChREBP) [12–15].

The analysis of lipid profiles in circulating erythrocyte membranes (EM) offers an extensive information on metabolic pathways involved in lipid metabolism and can be utilized to investigate the relationship between the patterns of fatty acid metabolism and disease [12, 13]. The fatty acids composition in EM may reflect the status of NAFLD and serve as a prognostic marker useful in monitoring the patients following various dietary regimens. Hodson et al. showed that docosahexaenoic acid was associated with improvements in hepatic metabolism and hepatic insulin sensitivity [14]. Notarnicola et al. revealed that saturation index in erythrocytes membrane was a helpful tool in the diagnosis and the staging of NAFLD [15]. In the current study we aimed to investigate the effect of 6-month dietary intervention on lipid profiles in EM of patients diagnosed with NAFLD.

## 2. Materials and Methods

### 2.1. Intervention

During this 6-month project, the patients were subjected to a dietary intervention in order to reduce the fatty liver. The protocol of the study has been accepted by the local bioethical committee at the Pomeranian Medical University in Szczecin and conducted in accordance with the principles expressed in the Declaration of Helsinki. Before and after the dietary intervention, the following measurements were performed among all of the patients: the assessment of the degree of fatty liver, anthropometric measurements, biochemical measurements, and the profile of fatty acids in erythrocyte membranes. 166 patients, hospitalized at the Clinic of Hepatology and Transplantology (CHT), Public Voievoid's Hospital in Szczecin, Poland, and diagnosed with NAFLD were enrolled in the study. Out of this number, 112 were further followed in the CHT's outpatient clinic throughout and till the end of the project. Of all patients enrolled, 54 dropped out due to not compliance to the following poor adherence to 72-h nutritional diaries, failure to reduce body weight and the degree of fatty liver, absence, and no shows-up at scheduled control visits.

### 2.2. Patients

The studied group consisted of 55 patients with NAFLD who, for a period of 6 months, followed a diet aiming at the reduction of the degree of fatty liver. The percentage of men in the group was 62%. The average age was 49 years (the range: 30-76 years old).

Every participant issued a written consent form to take part in the study and was informed about its course, benefits, and potential side effects. The main exclusion criterion was alcohol consumption of the amount above 20g of ethanol per 24h for both men and women. The patients who qualified for the study did not take any medicine that could significantly influence lipid and glucose metabolism (fibrates, statins, metformin, and corticosteroids). Additional exclusion criteria were carrier of HBV (hepatitis B virus) or HCV (hepatitis C virus), high level of physical activity (>3000 kcal/week during leisure time), the change in physical activity during the dietary intervention, vegetarianism or the application of special nutritional recommendations, and noncompliance.

### 2.3. The Evaluation of the Degree of Fatty Liver

The degree of fatty liver was estimated on the basis of ultra sonography. The analysis was carried out twice, before and after the period of dietary intervention. The degree of fatty liver was estimated on the basis of the Hamaguchi scale [16] by means of a high resolution scanner B-mode (Acuson X300). This method evaluates the severity of the illness in a 4-degree scale. The validation of the Hamaguchi scale made it possible to determine the sensitivity of the method at the level of 91.7% and its specificity at 100%.

### 2.4. Dietary Recommendations

The diet was adjusted to the individual calorie needs of every patient. Protein consumption was determined at the level of 1.0 g/kg of body mass/24h. Over half of the amount of protein was of animal origin, originating in dairy products and fish. The need for vitamins was covered by the presence of a wide range of fruit and vegetables, adjusted in such a way to ensure the proper functioning of liver enzymatic systems, especially in terms of vitamins A, K, C, and the B family. Fruit and vegetables were largely recommended in raw form, rich in nondigested food remains (soluble and insoluble fractions of nutritional fibre). Fibre in every diet variant was maintained at the level of 30-35g/24h. The amount of minerals was maintained at the physiological level. The supply of sodium provided via kitchen salt was reduced to the amount of 5g/24h. The recommended fats were mainly of plant origin, with olive oil and rapeseed oil as main sources. The energy originating from the supply of fat was between 20-30% of the total energy need of the organism. In relation to carbohydrates, the supply was 55-65%. The main sources of carbohydrates include complex products as well as the least processed ones, such as wholemeal bread, wholegrain pasta, thick groats, and natural rice. Omega-3 and omega-6 fatty acids were recommended in the doses that were in accordance with the reference consumption levels for the Polish population described in the Nutritional Norms of the National Food and Nutrition Institute.

### 2.5. The Isolation of Erythrocyte Membranes from Peripheral Blood

Blood in the amount of 10 ml was collected to the vials with heparin and then centrifuged (1850 x g, 4°C, 10 min), separating plasma from blood cells. 0.5 ml of blood cells was collected into a plastic test-tube and 10 ml of buffered sodium chloride solution (PBS) was added. The blood cells were washed 3 times until the supernatant was transparent. 13 ml of distilled water and 200 ul of BHT were added to the washed blood cells. In order to haemolyse the blood cells, the whole solution was incubated for 5 min at – 80°C. Subsequently, the blood cells were centrifuged for 60 min (1850 g, 4°C) and the supernatant was removed [3]. The erythrocyte membranes were stored at – 80°C until they were isolated.

### 2.6. The Isolation and the Analysis of Fatty Acids

Erythrocyte shadows isolated from 0.5 ml of peripheral blood were extracted by means of the modified Folch method [17]. The analysis of the fatty acid profile was conducted using gas chromatography (Agilent Technologies 7890A GC System). A capillary column (Supelcowax™ 10 Capillary GC Column, Supelco, Bellefonte, PA, USA) with the size of 15 m x 0.10 mm, 0.10 *μ*m was used in the study. The conditions of chromatographic separation were as follows: the initial temperature was 60°C (0 min); next, it rose by 40°C/min until it reached 160°C (0 min); the next increase in temperature was 30°C/min until it reached 190°C (0.5 min). During the next stage, the increase was also 30°C/min and it lasted until the temperature reached 230°C (2.6 min). The temperature of 230°C was maintained for 4.6 min. The whole analysis lasted about 8min. Hydrogen, which is a carrier gas, was flowing through the column with the speed of 0.8 ml/min.

### 2.7. The Statistical Analysis

The statistical analysis was performed using the “R 3.0.2” computer program. In order to check the normal distribution, the Shapiro-Wilk test was used. Because the distribution did not deviate from the norm, parametric tests were used in the calculations. The results are presented as mean values and standard deviation (SD). In order to check the differences between the studied parameters before and after the application of the diet, the Student test (t-test) was used for the paired data. In order to estimate the correlation, the Pearson's correlation test was used. The values of p<0.05 were considered as statistically important. The values being at the threshold of statistical significance were established at p<0.055.

With reference to the results which were not statistically significant, the abbreviation NS (not significant) was used instead of p.

## 3. Results

### 3.1. The Differences in Body Content and the Anthropometric Parameters before and after the Dietary Intervention

During the 6-month dietary intervention in patients from the studied group, statistically significant reduction in the degree of fatty liver, body mass, BMI, fat mass, and water content was observed. The dietary regimen was not associated with the change of fat-free body mass content in this group (Table 1).

### 3.2. The Differences in Biochemical Parameters before and after the Dietary Intervention

The patients who followed the diet also significantly improved their basic biochemical parameters in the blood: AST, ALT, total cholesterol, HDL, insulin concentration, and insulin resistance (Table 2).

### 3.3. The Differences in the Fatty Acid Profile before and after the Dietary Intervention

The patients from the studied group expressed significant differences in terms of the content of key fatty acids in erythrocyte membranes. After the 6-month diet, the following types of fat content was reduced: palmitoleic acid (C16:1), stearic acids (C18:0n9), oleic acid (C18:1), linoleic acid (LA), and arachidonic acid (AA). Furthermore, the increase in the level of docosahexaenoic acid (DHA) was also observed. The differences in the levels of palmitic acid (C16:0) and eicosapentaenoic acid (EPA) were at the threshold of statistical significance. The results of patients from the studied group are presented in Table 3.

### 3.4. The Differences in the Content of Particular Fatty Acid Groups and in the Indexes of Fatty Acids

The patients from the studied group increased the value of omega-3 index, which is a percentage sum of the content of EPA and DHA labelled in erythrocyte membranes. Decreased concentrations of SFA and increased concentrations of PUFA were also observed. The results of the studied group are presented in Table 4.

### 3.5. The Results of the Correlation of Fatty Acids with the Biochemical and Anthropometric Parameters

The body mass and BMI were positively correlated with stearic acid (0.286, p<0.05). ALA was negatively correlated with the important factors of NAFLD progression, insulin resistance (-0.426, p<0.05) and ALT (-0.410, p<0,05).

The parameter expressing the highest correlation turned out to be total cholesterol, which was positively correlated with SFA (0.275, p<0.05) and MUFA (0.239, p<0,05) and negatively correlated with PUFA (0.339, p<0,05). The LDL fraction cholesterol expressed a positive correlation with palmitic (0.431, p<0.05), stearic (0.369, p<0.05), oleic (0.193, p<0.05), and arachidonic acids (0.186, p<0.05). The HOMA-IR index was negatively correlated with ALA (-0.426, p<0.05), the omega-3 index (-0.346, p<0.05), and the content of PUFA (-0.361, p<0.05).

## 4. Discussion

The profile of fatty acids present in human blood is a resultant of the consumption of dietary lipids, the activity of the lipolytic adipose tissue, and the biosynthesis of fatty acids [18]. The results of the present study show that the 7% weight (average 6.6 kg) loss has been associated with the significant reduction of the degree of liver steatosis and improvement in biochemical parameters. Among those patients, a significant reduction of levels of AST, ALT, and insulin and in insulin resistance (expressed by the HOMA-IR index) was observed. Changes in the concentration of cholesterol and insulin significantly influence the pathways associated with the synthesis and metabolism of fatty acids. Permanent hyperinsulinemia and insulin resistance in NAFLD patients, cause a situation in which the % contribution of fatty acid biosynthesis may increase 5 or 6 times with reference to physiological conditions [7].

Among patients from the studied group we observed a trend towards reduction of the palmitic acid level as well as significant decrease of the stearic acid. These changes might have originated from both the dietary intervention and* de novo* lipogenesis. The characteristic feature of the profile of fatty acids of EM was the reduction of the content of all saturated acids (Table 5), which might have been associated with the significant elimination of food sources of these acids among patients following the diet. Significant changes in the profile of fatty acids also encompassed the reduction of palmitoleic and oleic acids. This phenomenon might be associated with the decrease conversion of palmitic acid into palmitoleic acid and stearic acid into oleic acid or the reduction in the supply of these acids in the diet. Patient menus assumed the exchange of the consumed SFA for MUFA and PUFA. It has to be pointed out that the type of fats recommended in the diet was mainly based on olive oil and rapeseed oil, which are an important source of oleic acid and palmitoleic acid [19]. The increased consumption of MUFA originating from plant oils should increase the content of C16:1 and C18:1 in EM. However, it turned out that the general content of MUFA did not change after the application of the diet (Table 5) and the contents of C16:1 and C18:1 were significantly lower (Table 4). The declared supply of MUFA recreated on the basis of the 72-hour nutritional diaries was over 40% of the energy originating from fats. Thus, a possible hypothesis is that the change in the fatty acid profile during the reduction of fatty liver was not only a consequence of dietary change.

One of the defensive mechanisms against the lipotoxicity of FFA is the conversion of saturated acids (C16:0, C18:0) into monounsaturated acids (C16:1, C18:1), which has been associated with the increased activity of desaturase [20]. Insulin and cholesterol (oxysterols) have been shown to the influence the activation of transcription factors from the ChREBP and SREBP-1c families, which were directly involved in* de novo *lipogenesis [21–26]. These two transcription factors play the main role in the activation of genes associated with the most important enzymes of change of fatty acid biosynthesis pathways, such as FAS, ELOVL 6, and SCD [27]. With the reduction in the activity of ChREBP and SREBP-1c, the expression of genes associated with the regulation of fatty acid biosynthesis pathway is also reduced. Similar observation were noticed in many other studies and summarized by Ferre [28]. Patients with more advanced form of the disease should have higher content of C16:1 and C18:1 in EM, and the reduction of fatty liver should be manifested by a gradual decrease in their content. Puri et al. observed that patients with simple steatosis or steatohepatitis had significantly increased total plasma MUFA level, driven by palmitoleic and oleic acids content. Moreover, palmitoleic acid, oleic acid, and palmitoleic acid to palmitic acid ratio were significantly increased in NAFLD across multiple lipid classes [29]. We observed this relation among our patients.

In EM of the patients from the studied group, we observed statistically significant increase in the general content of polyunsaturated fatty acids. PUFA play an important role in the regulation of fatty acid biosynthesis because they are the inhibitors of the expression of enzymes present during these processes [30, 31]. PUFA have an antagonistic effect in relation to insulin and cholesterol, which might inhibit the effect of activation of these factors during the progression of NAFLD. [32–36]. The changes of PUFA in EM included a significant increase in the concentration of EPA (at the threshold of statistical significance – p<0.055) and DHA, and a decrease in the content of ALA and AA. It was interesting to see that among the patients following the diet, there was an increase in the concentration of acids with a generally anti-inflammatory effect (EPA and DHA) and a decrease in relation to acids which are the precursors of proinflammatory derivatives (LA and AA) [37–47]. Puri et al. showed that patients with NAFLD have higher level of proinflammatory fatty acid mediators (HETE). The increase in lipoxygenase metabolites - 5(S)-hydroxyeicosatetraenoic acid (5-HETE), 8-HETE, and 15-HETE is characteristic for progression from normal to simple steatosis, to NASH [29]. The increase in the concentration of EPA and DHA was also visible in the case of the improvement of the omega-3 index parameter, which is helpful in the estimation of the risk of occurrence of circulatory system illnesses. Von Schacky et al. showed that average omega-3 index in the general population of Kansas City (US) was estimated in the level of 4.9%, in Germany 5.6%, in Japan 8.5% and in Korea 11%[48]. The value of the omega-3 index before the beginning of the dietary intervention was 1.92%, whereas after the diet it was 4.12%. On average, the level of EPA and DHA doubled among patients who followed the diet, which lead to a decreased risk of occurrence of cardiovascular incidents [48]. Moreover, Hondson et al. revealed that individuals who achieved a change in erythrocyte DHA enrichment *⩾*2% show favourable changes in hepatic fatty acid metabolism and insulin sensitivity [14]. Our findings also confirmed this observation.

The study assumed the analysis of particular types of fatty acids during the regression of the illness associated with the dietary intervention. The correlation analysis showed that some acids were strongly related to NAFLD, even in the case of a lack of a direct relation to the degree of fatty liver. The biggest focus should be on omega-3 fatty acids. Despite the fact that in patients following the dietary regimen no change in the concentration of ALA was observed, its correlations with the pathophysiological factors of NAFLD turned out to be the most significant. It also appeared that this acid negatively correlated with ALT and HOMA-IR, factors implicated in the pathogenesis of NAFLD. The correlation coefficients in both cases were the highest among analyzed dependencies and indicated a possible relation between the level of ALA and the progression of the disease. The omega-3 index also had a negative correlation in relation to the insulin resistance parameter, and the general pool of PUFA in EM was significantly related to the level of total cholesterol and insulin resistance. This highlights the role of PUFA, especially of the omega-3 family of acids, in the NAFLD pathogenesis. As evidence in already conducted studies, the supply of PUFA in NAFLD patients is lower than in the healthy population, regardless of the degree of fatty liver and the omega-3 supplementation was associated with the reduction of fatty liver as well as the decrease of ALT, AST, cholesterol, and insulin resistance [49]. Cussons et al. showed that the short-term supplementation with omega-3 acids at a dose of 4g/24h significantly reduced the degree of fatty liver [50].

## 5. Conclusion

Our study has limitations, which are (i) lack of liver biopsy and histopathologic assessment of steatosis/steatohepatitis and (ii) the limited dietary evaluation based on 72-h food diaries only. Nevertheless the profile of fatty acids turned out to be a sensitive surrogate marker reflecting of potentially pathological changes in the fatty liver of NAFLD patients following dietary management. Starting from these evidences, our findings reveal the importance and potential clinical relevance of lipidomic analysis in the management of NAFLD.

## Figures and Tables

**Table 1:** Dietary recommendations and current consumption according to 72-h diaries.

**Parameters**	**Recommended value**	**72-h diaries**
		**(after 6-months)**
Average Calorie intake [kcal]	1840 ± 537	1760 ± 421
Protein [%]	15	17,1 ± 5,3
Fat [%]	20-30	19,3 ± 7,2
SFA [%]	10	34,2 ± 9,9
MUFA [%]	60	40,7 ± 7,7
PUFA [%]	10	25.1 ± 6.6
Carbohydrates [%]	55-65	63,6 ± 8,9
Fiber [g/day]	30-35	31,6 ± 6,5

**Table 2:** The differences in body content and the anthropometric parameters before and after the dietary intervention. BMI: body mass index.

**Parameters**	**Before diet**		**After diet**		**p. value**
	**Mean **	**SD**	**Mean **	**SD**	
Age [years]	49.39	13.28	49.37	13.28	NS
**Stage of steatosis (1-4)**	**2.54**	**0.86**	**1.14**	**0.89**	**p<0.01**
**Body mass [kg]**	**94.64**	**18.89**	**88.03**	**17.71**	**p<0.01**
**BMI [kg/m** ^**2**^ **]**	**32.59**	**5.28**	**29.92**	**5.31**	**p<0.01**
**Fat mass [kg]**	**36.53**	**9.96**	**31.34**	**9.57**	**p<0.01**
**Fat content [**%**]**	**38.64**	**6.48**	**35.57**	**6.85**	**p<0.01**
Fat-free body mass [kg]	57.55	12.74	56.33	12.19	NS
**Water content [kg]**	**43.09**	**8.92**	**41.94**	**8.48**	**p<0.05**

**Table 3:** The differences in biochemical parameters before and after the application of the diet.

**Biochemical parameters**	**Before diet**		**After diet**		**p. value**
	**Mean **	**SD**	**Mean**	**SD**	
**Aspartate transaminase (AST) [U/L]**	**30.76**	**20.20**	**26.16**	**20.63**	**p<0.01**
**Alanine transaminase (ALT) [U/L]**	**49.15**	**29.99**	**33.35**	**22.81**	**p<0.01**
Gamma-glutamyltransferase (GGTP) [U/L]	123.20	358.95	136.42	443.43	NS
Triacylglycerols (TG) [mg/dl]	160.00	237.52	129.03	86.45	NS
**Total cholesterol [mg/dl]**	**214.67**	**49.39**	**201.62**	**42.99**	**p<0.05**
**High density lipoprotein (HDL) [mg/dl]**	**50.83**	**13.75**	**56.71**	**16.46**	**p<0.05**
Low density lipoprotein (LDL) [mg/dl]	132.13	64.92	122.47	38.29	NS
Total lipids [mg/dl]	695.47	317.50	657.83	157.30	NS
Fasting glucose [mg/dl]	104.29	26.25	101.62	21.05	NS
**Fasting insulin [U/ml]**	**25.07**	**17.33**	**9.93**	**9.30**	**p<0.05**
**HOMA-IR**	**5.54**	**11.65**	**2.63**	**2.44**	**p<0.05**

**Table 4:** The differences in the fatty acid profile before and after the dietary intervention.

**Fatty acid [**%**]**	**Before diet**		**After diet**		**p. value**
	**Mean **	**SD**	**Mean **	**SD**	
C10:0 capric acid	2.05	3.13	2.37	0.72	NS
C14:0 myristic acid	2.18	2.15	2.35	0.99	NS
C14:1 myristoleic acid	0.75	0.72	1.38	5.30	NS
C15:0 pentadecanoate acid	0.29	0.32	0.27	0.24	NS
**C16:0 palmitic acid**	**35.81**	**11.22**	**34.98**	**11.17**	**p<0.055**
**C16:1 palmitoleic acid**	**1.04**	**0.69**	**0.60**	**0.63**	**p<0.05**
C17:0 heptadecanoic acid	0.49	0.32	1.63	5.51	NS
C17:1 heptadecanoic acid	0.54	0.64	0.58	0.63	NS
**C18:0 stearic acid**	**30.53**	**10.28**	**28.90**	**9.86**	**p<0.05**
**C18:1 n9 oleic acid**	**7.22**	**6.63**	**5.95**	**5.13**	**p<0,05**
C18:1 n7 vaccenic acid	0.71	0.62	0.59	0.56	NS
**C18:2 n6 linoleic acid (LA)**	**4.98**	**3.50**	**4.27**	**3.67**	**p<0.05**
C18:3 n6 *γ*- linolenic acid (GLA)	0.22	0.31	0.32	0.59	NS
C18:3 n3 *α*- linolenic acid (ALA)	0.70	0.98	0.96	0.76	NS
C20:0 arachidic acid	0.35	0.37	0.49	0.70	NS
C20:1 eicosanoic acid	0.35	0.27	0.41	0.38	NS
C20:3 n6 eicosatrienoic acid	0.94	0.64	0.91	0.72	NS
**C20:4 n6 arachidonic acid (AA)**	**6.22**	**6.67**	**5.10**	**5.77**	**p<0.05**
**C20:5 n3 eicosapentaenoic acid (EPA)**	**0.57**	**0.53**	**0.77**	**0.68**	**p<0.055**
C23:0 tricosanoic acid	0.27	0.51	0.25	0.50	NS
C22:4 n6 docosatetraenoic acid	1.14	1.22	1.52	1.22	NS
C22:5 n3 docosapentaenoic acid	1.20	1.21	1.94	1.36	NS
**C22:6 n3 docosahexaenoic acid (DHA)**	**1.34**	**2.35**	**3.34**	**2.36**	**p<0.05**

**Table 5:** The differences in the content of particular fatty acid groups and in the indexes of fatty acids.

**Parametr [**%**]**	**Before diet**		**After diet**		**p.valeu**
	**Mean **	**SD**	**Mean **	**SD**	
**Omega-3 index**	**1.92**	**2.62**	**4.12**	**2.82**	**p<0.05**
**SFA**	**73.00**	**20.08**	**70.28**	**20.77**	**p<0.05**
MUFA	10.84	7.51	10.25	11.69	NS
**PUFA**	**16.14**	**14.59**	**19.46**	**13.90**	**p<0.01**

## Data Availability

Patients data include in manuscript are available in Department of Biochemistry and Human Nutrition, Pomeranian Medical University in Szczecin.
